# Ecological Momentary Assessment Is a Feasible and Valid Methodological Tool to Measure Older Adults’ Physical Activity and Sedentary Behavior

**DOI:** 10.3389/fpsyg.2018.01485

**Published:** 2018-08-15

**Authors:** Jaclyn P. Maher, Amanda L. Rebar, Genevieve F. Dunton

**Affiliations:** ^1^Department of Kinesiology, University of North Carolina at Greensboro, Greensboro, NC, United States; ^2^Department of Preventive Medicine, University of Southern California, Los Angeles, CA, United States; ^3^School of Health, Medical and Applied Sciences, Central Queensland University, Rockhampton, QLD, Australia

**Keywords:** exercise, sitting, aging, intensive longitudinal data, measurement

## Abstract

Ecological momentary assessment (EMA) has the potential to yield new insights into the prediction and modeling of physical activity (PA) and sedentary behavior (SB). The objective of this study was to determine the feasibility and validity of an EMA protocol to assess older adults’ PA and SB. Feasibility was determined by examining factors associated with EMA survey compliance and if PA or SB were impacted by EMA survey compliance. Validity was determined by comparing EMA-reported PA and SB to objectively measured PA and SB at the EMA prompt. Over 10 days, older adults (*n* = 104; Age_range_ = 60–98 years) received 6 randomly prompted EMA questionnaires on a smartphone each day and wore an ActivPAL activity monitor to provide a device-based measure of PA and SB. Participants reported whether they were currently engaged in PA or SB. Older adults were compliant with the EMA and ActivPAL protocol on 92% of occasions. Differences in EMA compliance differed by weight status. Among overweight and obese older adults EMA compliance differed by sex (OR = 3.15, 95% CI: 1.43, 6.92) and day of week (OR = 1.79, 95% CI: 1.33, 2.41). Among normal weight older adults, EMA compliance differed by time of day (OR = 1.52, 95% CI: 1.02, 2.30). EMA compliance did not differ for device-based PA or SB in the 15 min before versus the 15 min after the EMA prompt, suggesting that these behaviors did not influence likelihood of responding and responding did not influence these behaviors (*p*s > 0.05). When PA was reported through EMA, participants engaged in less device-based PA in the 15 min after compared to the 15 min before the EMA prompt (*p* = 0.01), suggesting possible reactance or a disruption of PA. EMA-reported PA and SB were positively associated with higher device-based PA and SB in the ±15 min, respectively, supporting criterion validity (*p*s < 0.05). The assessment of older adults’ PA and SB through EMA is feasible and valid, although there may be PA reactance to EMA prompting. Therefore, EMA represents a significant methodological tool that can aid in our understanding of the environmental, social, and psychological processes regulating older adults’ PA and SB in the context of everyday life.

## Introduction

Physical activity (PA) and sedentary behavior (SB) independently contribute to health and well-being across the lifespan (Physical Activity Guidelines Advisory Committee, 2008; de Rezende et al., 2014). Yet, PA levels decline and SB levels increase as individuals age, with older adults engaging in less than 10 min of moderate- to vigorous-intensity PA each day and sitting for more than two-thirds of their waking hours (Harvey et al., 2014; Martin et al., 2014).

To understand these behaviors as well as their causes and correlates, researchers rely on accurate and unbiased measures of PA and SB (Haskell, 2012). Retrospective self-report measures are commonly employed to assess these heath behaviors despite well-documented errors and systematic biases associated with these measures (e.g., inaccurate recall, social desirability; Adams et al., 2005; Prince et al., 2008). Device-based measures (e.g., accelerometers, pedometers) are employed to overcome many of the recall-based limitations of retrospective measures as device-based measures are collected in real-time and provide an objective measure of behavior. However, device-based measures are unable to specify the type of activity or contextual factors (e.g., physical, social, temporal, affective) surrounding these behaviors which are important factors to consider when developing interventions (Troiano et al., 2012). Additionally, device-based measures are susceptible to high amounts of missing data resulting from device non-wear (Colley et al., 2010).

These methodological weaknesses may be addressed through recent advancements in mobile technologies, which can collect information on PA and SB through a real-time, self-report data capture strategy such as Ecological momentary assessment (EMA) (Dunton, 2017). EMA studies are designed to repeatedly and intensively sample a persons’ behaviors, cognitions, affect, context, and other experiences in real-time in the person’s natural environment (Stone and Shiffman, 1994). Software applications can be loaded onto a mobile phone to execute an EMA protocol, alerting participants to complete a brief electronic questionnaire at random times throughout the day to better understand the phenomenon of interest as it naturally occurs in the real world, in real time. Therefore, EMA has the potential to yield new insights into the prediction and modeling of PA and SB.

Advances in mobile technology over the past decade provide an optimal environment in which EMA studies of PA and SB can be conducted. The vast majority of Americans own a mobile phone (95%) and nearly three-quarters of Americans own a smartphone (77%) (Pew Research, and Center, 2017). Even mobile technology adoption among older adults is occurring at high rates with 80% of older adults owning a mobile phone and 42% of older adults owning a smartphone (Pew Research, and Center, 2017).

As mobile phone and smartphone technology become more ubiquitous, the opportunities for using EMA to assess PA and SB have increased across various populations. In fact, several studies have documented the feasibility, validity, and utility of EMA, through smartphone applications, to measure PA and SB in children, adolescents, and adults (e.g., Rofey et al., 2010; Dunton et al., 2011, 2012; Knell et al., 2017). Among adults age 50 years and older, the use of EMA to assess activities of daily living, including PA, has primarily been conducted via paper and pencil daily diaries (Cain et al., 2009). For instance, in a 7-day daily diary study of older adults (age_range_ = 69–92), Stel et al. (2004) found that paper and pencil end of day reports regarding the duration of PA showed acceptable correspondence with the Longitudinal Aging Study Amsterdam Physical Activity Questionnaire, a population-level, recall-based measure of PA. However, there has yet to be a study examining signal-contingent EMA via mobile phones exclusively among older adults as a tool to measure PA and SB. Because the activity profiles as well as adoption and integration with mobile technology among older adults differs from younger segments of the population (Dai et al., 2015; Pew Research, and Center, 2017), it is unclear if EMA is a feasible and valid methodological tool to assess PA and SB among older adults.

Therefore, a 10-day EMA study was conducted to determine the feasibility and validity of a real-time EMA protocol using brief, self-reported electronic questionnaires on smartphones to measure older adults’ current PA and SB in the real world. Importantly, this EMA study was not designed to measure total volume of PA or SB. The first objective was to determine whether time-varying and time-invariant factors influenced EMA response rates. The second objective was to determine whether concurrent PA or SB were associated with EMA survey non-response. This aim examined whether being engaged in PA or SB prevented older adults from responding to EMA prompts. The third objective was to determine whether the act of completing the EMA survey lead to changes in PA or SB after the EMA prompt. This aim examined whether there was behavioral reactance to the EMA survey prompt or whether it interrupted behavior. The fourth objective was to examine the criterion validity of EMA-reported activity levels by comparing with device-based PA and SB measured through an ActivPAL activity monitor.

## Materials and Methods

### Participants and Recruitment

Community-dwelling older adults from Los Angeles County were recruited. Recruitment occurred through announcements at local senior centers and retirement communities as well as through a subject pool of adults that had previously participated in research studies at a major university in Southern California. Inclusion criteria consisted of the following: (a) age 60 years or older and (b) living in Los Angeles County. Older adults were excluded if they (a) did not speak/read English fluently, (b) had any functional limitations that prevent standing or walking on their own or seeing/utilizing a smartphone’s basic functions, (c) had been diagnosed as having dementia or Alzheimer’s Disease.

### Procedures

Eligible older adults attended an introductory appointment, at which participants were familiarized with the study procedures and equipment and provided informed consent. Participants were assigned a MotoG4 smartphone and ActivPAL activity monitor. Participants were trained on how to use the mobile phone to answer brief electronic questionnaires assessing self-reported behavior. EMA questionnaires were delivered on the mobile phone through the commercially available, android-based EMA application, movisensXS (Karlsruhe, Germany). During the training, participants completed a sample EMA questionnaire on the mobile phone.

Participants were instructed to carry the mobile phone with them for the duration of the 10-day study. Participants were prompted on the mobile phone six times per day to complete EMA questionnaires, with each prompt occurring randomly within one of six preprogrammed, 2-h windows between 8:00am and 8:00pm. The phone rang to signal an EMA prompt. Upon hearing the auditory signal, participants were instructed to stop their current activity and complete the EMA question sequence. This process required 2–3 min. If participants were driving or engaged in another incompatible activity, participants were instructed to ignore the prompt. If an EMA questionnaire was not completed after the initial prompt, the phone emitted up to three reminder signals at 5-min intervals. Following the third reminder, the EMA questionnaire became inaccessible until the next questionnaire.

Participants were also instructed to wear the ActivPAL activity monitor on their anterior thigh during all sleeping and waking hours. Activity monitors were waterproofed using a nitrile sleeve and athletic tape to allow for participants to wear the activity monitor while showering; however, participants were instructed to remove the activity monitor when it would be submerged under water (e.g., bath, swimming). Participants were provided two logs to indicate (1) any times when the activity monitor was removed and (2) daily wake and sleep times to facilitate the differentiation of activities completed in a seated or reclined position during sleeping and waking hours.

Participants completed a paper and pencil questionnaire which assessed demographic information. Following the conclusion of the introductory session, the study protocol began immediately. During the protocol, participants received one phone call or email from the research staff to remind them of the study procedures and inquire about any technical problems. Participant compensation was prorated based on compliance, with participants answering at least 80% of the EMA prompts earning the full $80. All study procedures were deemed to be ethical and approved *a priori* by the local Institutional Review Board. A primary ethical concern in this study was protecting participants’ privacy as their responses to EMA on the mobile phone were wirelessly transmitted to a server and stored on the server following completion of the study. To help to ensure participants’ privacy in this study, the data collected was coded and contained no identifying information.

### Measures

#### EMA Self-Reported Behavior

**FIGURE 1:**
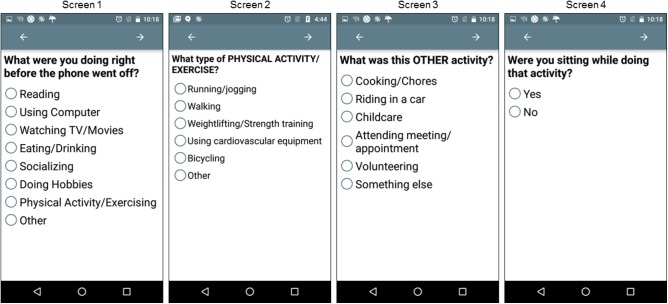
Ecological momentary assessment (EMA) screenshots. Images display how EMA items and response choices appeared on the display screen of the mobile phone. Only one response could be selected per screen. If a participant responded “Physical Activity/Exercising” on Screen 1, the participant was automatically directed to Screen 2. If a participant responded “Other” on Screen 1, the participant was automatically directed to Screen 3. On all occasions participants selected a response other than “Physical Activity/Exercising” on Screen 1, participants received the follow-up question on Screen 4.

Self-reported behavior was assessed through four questions during the EMA protocol. Initially participants responded to the item, “What were you doing right before the phone went off?” Participants were instructed to select whatever they considered the main activity, as participants could only select one response. Participants’ response to this initial question triggered three automatic branching questions. This first branching sequence was triggered if a participant responded, “Physical Activity/Exercising.” The branching follow-up question asked the participant, “What type of PHYSICAL ACTIVITY/EXERCISE?” The second branching sequence was triggered if participants responded, “Other,” to the initial question and subsequent branching asked participants, “What was this OTHER activity?” The third branching sequence was triggered when participants responded to the initial question with any response other than, “Physical Activity/Exercise.” This branching follow-up question asked the participant, “Were you sitting while doing that activity?” By definition, if an individual is engaged in PA or exercise at a given moment they cannot also be engaged in SB at that same moment even if they are sitting (Sedentary Behaviour, and Research Network, 2012). For example, bicycling on a recumbent bike occurs in a seated position; however, this activity produces greater than minimal amounts of energy expenditure so it cannot be classified as SB. EMA screen shots are displayed in **Figure 1**. EMA responses in which participants indicated the current activity was “Physical Activity/Exercise” were coded as *PA*. EMA responses in which participants indicated they were sitting while engaged in the current activity were coded as *SB*.

#### Device-Measured Physical Activity and Sedentary Behavior

The ActivPAL activity monitor provided a device-based measure of PA and SB. The ActivPAL activity monitor has been shown to a valid and reliable measures of older adults’ posture (e.g., sitting, standing) and movement (e.g., walking) (Grant et al., 2006, 2008; Kozey-Keadle et al., 2011). The ActivPAL activity monitor uses a proprietary algorithm to classify time spent sitting or lying, standing and stepping. ActivPAL activity monitor data was collected in 15-s epochs and time-stamped to facilitate linking with time-stamped EMA data. Only activity monitor data in the 15 min before and 15 min after each EMA prompt were included in this study. Participant activity monitor and sleep and wake time logs were used to determine periods of non-wear during waking hours. Occasions when participants indicated that they were not wearing the ActivPAL activity monitor in the 15 min before and 15 min after the EMA prompt were considered accelerometer non-wear and excluded from analysis. Additionally, if participants indicated in their sleep and wake time log that they were sleeping during the 15 min before and 15 min after the EMA prompt, data were excluded from the analysis to align with the definition of SB that it is a waking behavior (Sedentary Behaviour, and Research Network, 2012). Given that older adults’ most common type of PA is walking (Dai et al., 2015), concurrent PA was operationalized as total time spent stepping in the 15 min before and 15 min after the EMA prompt. Concurrent SB was operationalized as total waking time spent sitting in the 15 min before and 15 min after the EMA prompt.

#### Demographic and Time Variables

Participants’ age, sex, ethnicity, and height and weight were self-reported through a paper-and pencil questionnaire. Based on the time-stamp, EMA prompts occurring from 8:00am to 11:59am were coded as “morning,” from 12:00pm to 3:59pm as “afternoon,” and from 4:00pm to 8:00pm as “evening.” Day of week data was coded into a dichotomous variable of weekday (reference group) or weekend day.

### Data Analysis

The modeling approach for this study utilized multilevel modeling to account for the nesting of occasions of data within people. Preliminary analyses, including calculating intraclass correlation coefficients (ICCs) (Hox, 2002) and design effects (McCoach, 2010), were conducted to determine the necessity of multilevel modeling. These preliminary analyses indicated key outcomes such as EMA non-response (answered versus unanswered), self-reported current PA and SB, and device-based concurrent PA and SB had ICCs ranging from 0.06 (self-reported current SB) to 0.29 (EMA non-response) and design effects ranging from 1.98 (self-reported current SB) to 4.21 (EMA non-response). All of which suggest that there is a degree of dependence among observations and by not accounting for that nested dependence through an approach such as multilevel modeling, the Type I error rate would be inflated (McCoach, 2010).

Regarding Objective 1, two multilevel (level 1 = occasions, level 2 = people) logistic regression models were estimated. The first regressed EMA non-response (answered versus unanswered) on time-invariant demographic factors (i.e., age, sex, race/ethnicity, and weight status; level 2) and time-varying temporal processes (i.e., day of week and time of day; level 1). The second regressed ActivPAL activity monitor non-wear (versus valid wear) at the EMA prompt on time-invariant demographic factors and time-varying temporal processes listed above. Regarding Objective 2, a multilevel logistic model regressed EMA non-response on concurrent device-based PA and SB (i.e., ±15 min around the EMA prompt). Regarding Objective 3, multilevel repeated measures models were tested to compare time spent engaged in PA and SB in the 15 min before the EMA prompt to the 15 min after the EMA prompt. The model comparing PA before and after the EMA prompt only included occasions where “Physical Activity/Exercising” was reported as the current activity, and the model comparing SB before and after the EMA prompt only included occasions where participants indicated they were sitting during the current activity. Regarding Objective 4, two multilevel linear regression models were estimated. In the first, concurrent (±15 min) device-measured PA was regressed onto a dichotomous variable with EMA-reported current activity type coded as either “Physical Activity/Exercising” versus not reporting “Physical Activity/Exercising” (i.e., selected a response option besides “Physical Activity/Exercising”). In the second, concurrent (±15 min) device-measured SB was regressed onto a dichotomous variable with EMA-reported current activity coded as either sitting (i.e., responded “Yes” when asked if they were sitting during their current activity) vs. not sitting (i.e., responded “No” when asked if they were sitting during their current activity). Additional versions of these multilevel models were estimated to determine differences in concurrent PA and SB across specific EMA-reported current activity categories. In the first set of these models, concurrent (±15 min) device-measured PA was regressed onto an 11-level categorical variable of EMA-reported current activity type. “Physical Activity/Exercising” served as the reference group for this categorical variable. In the second set of models concerning SB, concurrent (±15 min) device-measured SB was regressed onto a 10-level categorical variable of EMA-reported current activity type. “Watching TV/Movies” served as the reference group for this categorical variable. For these additional models, pairwise contrast comparisons were conducted between all pairs.

All models were initially stratified by weight status (normal weight versus overweight/obese) to account for differences in activity patterns by weight status (Spees et al., 2012). If no differences by weight status were found, models were run for the entire sample with weight status included as a covariate. Sex, age, and ethnicity (Hispanic versus non-Hispanic) were included as covariates. Fixed-effects for all predictor variables were estimated in each model. A random intercept was also included in all models to account for the systematic influence of participants’ repeated observations. All modeling was conducting in Stata 14.

## Results

### Data Availability

**FIGURE 2:**
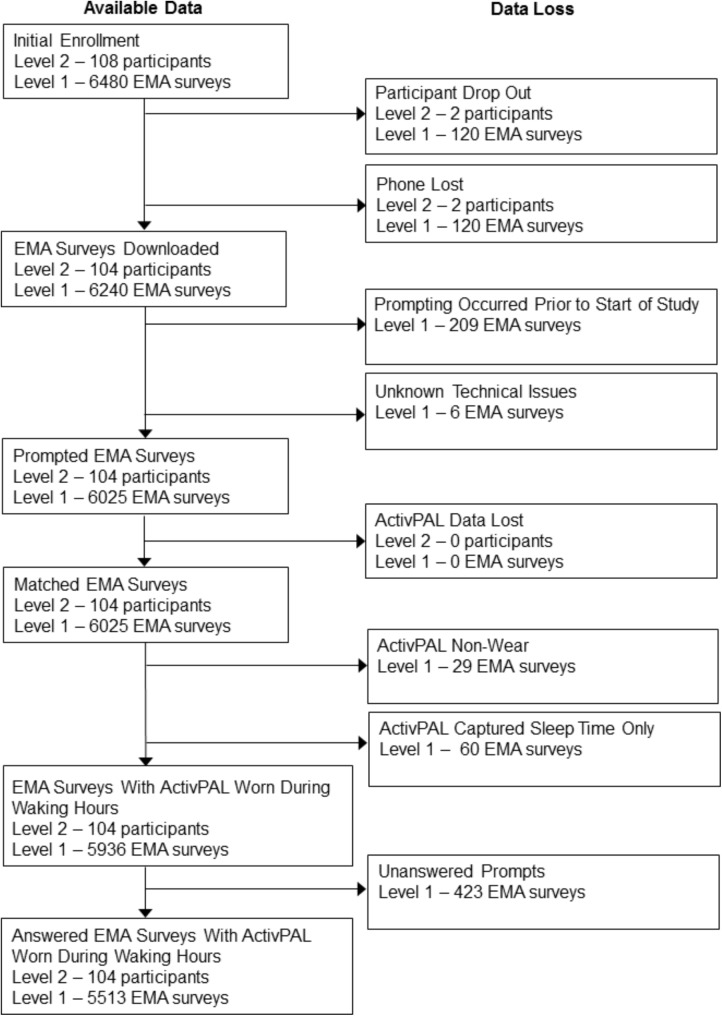
Flow chart of data availability. Level 1 represents the number of electronic EMA surveys, and Level 2 represents the number of participants. EMA Surveys Downloaded – the number of EMA surveys successfully downloaded from the mobile phone. Prompted EMA Surveys – the number of EMA surveys with a time and date record of being prompted. Matched EMA Surveys – the number of prompted EMA surveys that could be time-matched to available ActivPAL data. EMA Surveys with ActivPAL Worn During Waking Hours – the number of prompted EMA surveys that could be time-matched to data indicating ActivPAL was worn during that period. Accelerometer wear was based off of activity monitor logs. Answered EMA Surveys with ActivPAL Worn During Waking Hours – the number of answered EMA surveys that could be time-matched to data indicating ActivPAL was worn during that period.

A flow chat displaying data availability and sources of missingness for the 108 older adults initially enrolled in the study is displayed in **Figure 2**. Two participants dropped out of the study due to a medical emergency (*n* = 1) or participant burden (*n* = 1). Additionally, two participants lost their phones while enrolled in the study. These participants were excluded from the analyses. They did not differ from the rest of the sample on any demographics (all *p’*s > 0.05).

Of the 6,240 programmed EMA surveys (60 surveys across 104 participants), 209 EMA surveys were not prompted due to the timing of the introductory session on Day 1. For instance, a participant who had their introductory session at 3:00pm missed any prompts randomly occurring between 8:00am and 3:00pm on Day 1. Six EMA surveys were not prompted due to technical problems. No ActivPAL activity monitors were lost during the study, and there were no activity monitor malfunctions while downloading data. ActivPAL activity monitors were not worn during 29 of the EMA prompts (across 10 people) based on activity monitor logs. Nearly three-fourths (73%) of participants indicated that they never removed the monitor during the study. An additional 60 occasions were excluded from data analysis because, based on the sleep and wake time log, participants received the EMA prompt while sleeping, awoke to complete the questionnaire, then went back to sleep after completing it. This resulted in invalid data as the ±15 min window around the EMA prompt included sleep time only. Of the 5,936 EMA prompts that were matched with data from a worn activity monitor, a total of 423 prompts were unanswered, resulting in an analytic sample size of 5,513 answered EMA surveys across 104 participants. Approximately two-thirds of the sample provided data on at least 52 occasions across the 10-day study (Median _#ofoccasions_ = 55, Mean _#ofoccasions_ = 53, *SD*_#ofoccasions_ = 6.2, Range = 11–60). Due to the controversial nature of *post-hoc* power analyses for multilevel modeling (e.g., Hoenig and Heisey, 2001), such an analysis was not conducted; however, previous adequately powered EMA research studies have attempted to answer similar research questions with similar sample sizes at the occasion- and person-level (e.g., Dunton et al., 2011, 2012; Maher and Conroy, 2016; Maher et al., 2016; Fritz et al., 2017).

### Descriptive Statistics

Demographic characteristics for the analytic sample (*n* = 104) are displayed in **Table 1**. The average age of participants was 72 years (*SD* = 7).

**Table 1:** Descriptive statistics for key variables in older adults.

	*n* (%)
Sex	
Male	39 (37.5%)
Female	65 (62.5%)
Ethnicity	
Hispanic/Latino	12 (11.5%)
Non-hispanic/Latino	92 (88.5%)
Race	
White/Caucasian	77 (74.0%)
African–American	8 (7.7%)
Asian	10 (9.6%)
Other	4 (3.8%)
Two or more races	5 (4.8%)
Weight status	
Underweight/Normal weight	43 (41.7%)
Overweight	33 (32.1%)
Obese	27 (26.2%)
Marital status	
Never married	9 (8.7%)
Partnered	5 (4.8%)
Married	50 (48.1%)
Separated/Divorced/Widowed	39 (37.5%)
Annual household income	
<$80,000	34 (32.6%)
≥ $80,000	49 (47.1%)
Chose not to answer	21 (20.3%)
Living arrangement	
Independently in home/Apartment	98 (94.2%)
Assisted living community	1 (1.0%)
Other	5 (4.8%)
Currently own smartphone	
Yes	79 (76.0%)
No	25 (24.0%)


*Marriage, weight status missing *n* = 1.*

Of the EMA surveys answered while wearing the accelerometer, PA was reported as the main activity in 5.7% of EMA surveys. When participants reported “Physical Activity/Exercising” through EMA the most common physical activities were comprised of “Walking” (41.8%), “Bicycling” (9.5%), “Weightlifting/Strength Training” (5.7%), “Using Cardiovascular Equipment” (6.6%), “Running/Jogging” (1.6%), and “Other” (34.8%). Participants reported engaging in SB in 62.7% of EMA surveys. When participants reported sitting through EMA the most common sedentary activities were “Using Computer” (22.3%), “Watching TV/Movies” (13.5%), “Driving/Riding in a Car” (12.8%), “Eating/Drinking” (10.7%), “Reading” (10.5%), “Socializing” (8.5%), “Attending a Meeting/Appointment” (5.3%), “Doing Hobbies” (2.4%), “Cooking” (0.7%) and “Other” (13.3%).

Bivariate correlations among EMA-reported current activity and concurrent accelerometer data ignoring within-individual clustering were estimated for descriptive purposes. EMA-reported PA was moderately and positively correlated with accelerometer-derived concurrent PA (*r* = 0.27) as was the correlation between EMA-reported SB and accelerometer-derived concurrent SB (*r* = 0.29).

### Factors Influencing Compliance With Study Procedures

On average, participants answered 92% (range 20–100%) of the EMA prompts. The average EMA compliance while wearing the activity monitor was 92%. Regarding Objective 1, among overweight and obese older adults, the likelihood of EMA prompt non-response did not differ as a function of time of day, age, or ethnicity. Overweight and obese older adults were more likely to miss an EMA prompt on weekend days compared to weekdays (OR = 1.72, 95% CI: 1.29, 2.29). Additionally, among overweight and obese older adults, females were more likely to miss an EMA prompt compared to males (OR = 2.75, 95% CI: 1.35, 5.63). Among normal weight older adults, the likelihood of EMA prompt non-response did not differ by day of week, sex, age, or ethnicity. However, normal weight older adults were more likely to miss an EMA in the afternoon (OR = 1.53, 95% CI: 1.02, 2.30) compared to the morning. **Table 2** displays associations between EMA compliance and time-invariant demographic factors (i.e., age, sex, race/ethnicity; level 2) and time-varying temporal processes (i.e., day of week and time of day; level 1).

**Table 2:** Multilevel logistic regression results predicting EMA compliance among normal weight and overweight/obese older adults.

	Predicting EMA compliance among normal weight older adults Odds Ratio (CI)	Predicting EMA compliance among overweight and obese older adults Odds Ratio (CI)
Intercept	0.10 (0.01, 3.70)	0.02 (0.01, 1.14)
Age	0.98 (0.93, 1.03)	1.00 (0.95, 1.05)
Sex	1.52 (0.67, 3.46)	2.75^∗^ (1.35, 5.62)
Ethnicity	1.67 (0.50, 5.48)	1.74 (0.59, 5.15)
Weekend	1.29 (0.89, 1.87)	1.72^∗^ (1.29, 2.29)
Afternoon	1.53^∗^ (1.02, 2.30)	0.96 (0.69, 1.33)
Evening	1.04 (0.67. 1.61)	0.92 (0.67, 1.28)


*Coefficients in the table represent fixed effects. Multilevel model predicting EMA compliance (answered vs. not answered) among normal weight adults contained 2,518 observations from 44 participants. Multilevel model predicting EMA compliance (answered vs. not answered) among normal weight adults contained 3,394 observations from 60 participants. CI = 95% Confidence Interval. ^∗^*p* < 0.05.*

Also pertaining to Objective 1, there were no differences in accelerometer non-wear by weight status as a result, likelihood of accelerometer non-wear was examined across the entire sample (with BMI included as a covariate). The likelihood of accelerometer non-wear did not vary as a function of day of week, sex, age, ethnicity, or weight status; however, older adults were less likely to have accelerometer non-wear in the afternoon (OR = 0.33, 95% CI: 0.11, 0.97) compared to the morning. **Table 3** displays associations between accelerometer compliance and time-invariant demographic factors (i.e., age, sex, race/ethnicity, BMI; level 2) and time-varying temporal processes (i.e., day of week and time of day; level 1).

**Table 3:** Multilevel logistic regression results predicting accelerometer compliance among older adults.

	Predicting accelerometer compliance among older adults Odds Ratio (CI)
Intercept	0.01 (0.00, 313.18)
Age	1.00 (0.83, 1.21)
Sex	0.99 (0.08, 11.07)
Ethnicity	0.85 (0.02, 36.10)
BMI	1.07 (0.84, 1.38)
Weekend	2.06 (0.84, 5.04)
Afternoon	0.33^∗^ (0.11, 0.97)
Evening	0.50 (0.19. 1.31)


*Coefficients in the table represent fixed effects. Multilevel model predicting accelerometer compliance (valid wear vs. non-wear) among older adults contained 5,894 observations from 104 participants. BMI, Body Mass Index. CI, 95% Confidence Interval. ^∗^*p* < 0.05.*

Regarding Objective 2, there were no differences in compliance with EMA prompts as a function of concurrent device-based PA (OR = 0.98, 95% CI: 0.95, 1.01) or SB (OR = 1.00, 95% CI: 0.99, 1.01). Concurrent device-based PA during the ±15 min surrounding each EMA prompt did not differ depending on whether the EMA prompt was answered (*M* = 2.94 min, *SE* = 0.13) or unanswered (*M* = 2.73 min, *SE* = 0.19). Similarly, concurrent device-based time spent sitting during the ±15 min surrounding each EMA prompt did not differ depending on whether the EMA prompt was answered (*M* = 18.88 min, *SE* = 0.38) or unanswered (*M* = 19.10 min, *SE* = 0.48). These results did not differ by weight status.

### EMA Responses and Behavioral Reactance

Regarding Objective 3, on occasions in which PA was reported as the main activity during the EMA prompt, participants engaged in less PA in the 15 min after the EMA prompt (*M* = 3.35 min, *SE* = 0.26) as compared to the 15 min before the EMA prompt (*M* = 3.99 min, *SE* = 0.36; β = -0.64, *p* = 0.01). On occasions when older adults indicated that they were sitting at the EMA prompt, the amount of time spent sitting in the 15 min before (*M* = 10.55 min, *SE* = 0.18) and after the EMA prompt (*M* = 10.36 min, *SE* = 0.12) did not differ (β = -0.19, *p* = 0.10).

### Criterion Validity of EMA Responses

**FIGURE 3:**
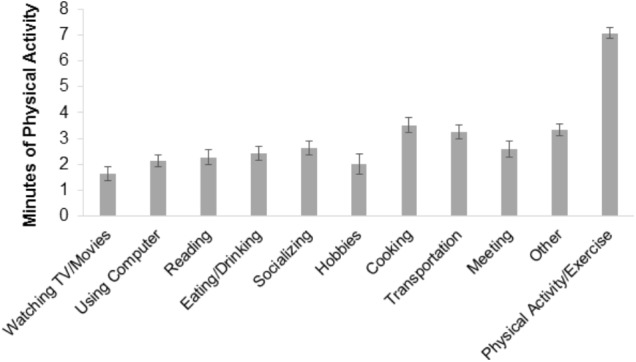
Device-based mean minutes of concurrent physical activity by EMA-reported current activity. Unadjusted estimates are displayed. Standard error (SE) bars are shown. Non-overlapping SE bars indicate a statistically significant difference between means at *p* < 0.01.

Regarding Objective 4, on occasions when participants responded to EMA prompts reporting “Physical Activity/Exercising,” their concurrent device-monitored PA was significantly greater (*M* = 7.09 min, *SE* = 0.21) compared to prompts where they did not report “Physical Activity/Exercising” (*M* = 2.69 min, *SE* = 0.12; *F* = 414.68, df = 1, *p* < 0.01). Pairwise contrast comparisons of concurrent device-based PA across the 11 possible EMA-reported current activities revealed that significantly more time was spent engaged in PA when EMA surveys reported “Physical Activity/Exercising” compared to all other self-reported activities (*p*s < 0.001; **Figure 3**).

**FIGURE 4:**
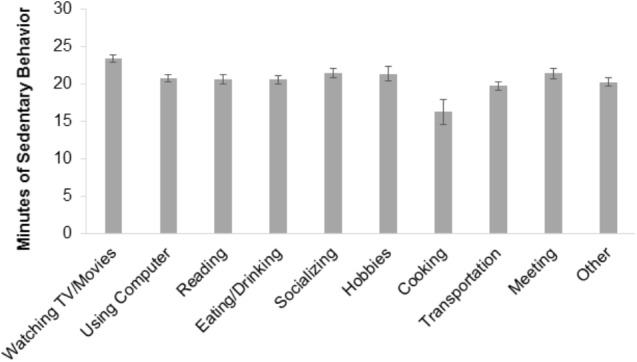
Device-based mean minutes of sedentary behavior by EMA-reported current activity. Unadjusted estimates are displayed. Standard error (SE) bars are shown. Non-overlapping SE bars indicate a statistically significant difference between means at *p* < 0.05.

Also regarding Objective 4, on occasion when participants responded to EMA prompts reporting sitting activities, their concurrent device-monitored SB was significantly greater (*M* = 20.91 min, *SE* = 0.37) compared to occasions when participants reported that they were not sitting (*M* = 15.66 min, *SE* = 0.26; *F* = 382.45, df = 1, *p* < 0.001). Regarding pairwise contrast comparisons of domain-specific types of SB, SB was significantly higher while watching TV/Movies compared to all other EMA-reported activities (*p*s < 0.05; **Figure 4**). SB was significantly lower while cooking/chores compared to all other EMA-reported activities (*p*s < 0.05). Additionally, SB was higher for EMA-reported sitting while using computer versus sitting while in a transit (*F* = 4.69, df = 1, *p* < 0.05), sitting while socializing versus sitting in transit (*F* = 7.25, df = 1, *p* < 0.01), and sitting in a meeting/appointment versus sitting in transit (*F* = 4.66, df = 1, *p* < 0.05).

## Discussion

As mobile phone technology becomes more ubiquitous, EMA has become a viable option to better understand patterns and contexts of PA and SB as well as the causes and correlates of these behaviors. While older adults’ adoption of mobile phone technology lags behind younger populations, previous studies suggest older adults are willing to engage with novel technology, and when adequately trained, can successfully implement it (Preusse et al., 2017). Results from this study suggest that among older adults, a 10-day EMA protocol designed to assess PA and SB with random, signal-contingent prompting occurring six times per day is both feasible and valid measures of PA and SB.

In the present study, older adults answered approximately 92% of EMA prompts received on the mobile phone. Older adults typically display high levels of compliance in EMA studies. A review by Cain et al. (2009) indicated that approximately 90% of EMA studies among older adults (e.g., within-day, signal contingent paper and pencil protocols, daily diary protocols) reported compliance rates of over 80%. More recently, EMA protocols designed to assess aspects of mood and activities of daily living delivered via mobile phone among older adults have also reported high levels of compliance (Fritz et al., 2017; Moore et al., 2016, 2017). However, compared to other studies employing signal-contingent EMA protocols to assess PA levels in children and adults, older adults displayed much higher levels of compliance (e.g., Dunton et al., 2011, 2012). Additionally, older adults were highly compliant with the ActivPAL activity monitor with 73% of participants never removing the activity monitor during the study protocol. Older adults’ adherence to this thigh-worn activity monitor protocol was greater than previous research employing a waist-worn activity monitor protocol in children or adults (Dunton et al., 2011, 2012). All of which suggests the EMA and ambulatory monitoring protocol employed in this study was not overly burdensome for older adults.

Regarding the first study objective, female older adults were more like than males to have missing EMA data. Perhaps more concerning, overweight and obese older adults were more likely to have missing EMA data on weekend days compared to weekdays; however, this trend was not present among normal weight older adults. Previous research regarding PA EMA protocols among children has indicated that children in a higher BMI percentile are less likely respond to EMA prompts compared to children with a lower BMI percentile (Dzubur et al., 2017). The fact that compliance patterns may differ by health-related characteristics such as weight status represents a possible area of concern for EMA research as individuals with higher BMI may be less likely to answer health-related questions. This could potentially limit researchers’ ability to explore differential mechanisms underlying (dis)engagement in health behaviors through EMA among individuals of different weight status. Future research may benefit from conducting focus groups to explore reasons why individuals do not answer EMA prompts and if those reasons differ by weight status.

Previous research investigating compliance among health behavior EMA protocols has indicted that ethnicity is significantly related to EMA compliance. Specifically this research has indicated that Hispanic/Latino individuals have lower compliance with EMA protocols compared to their white counterparts (Dunton et al., 2011; Dzubur et al., 2017). Such ethnic differences in EMA compliance did not emerge in this study, although only a small portion of the sample in the present study identified as Hispanic/Latino.

Among normal weight older adults, EMA data was more likely to be missing in the afternoons compared to mornings. Additionally, activity monitor non-wear was more likely to occur in the mornings compared to the afternoons. Previous EMA studies employing ambulatory monitoring via an activity monitor in adults and children have also documented higher rates of non-wear or non-valid activity monitor data surrounding the EMA prompt in the morning compared to the afternoon or evening (Dunton et al., 2011, 2012). Furthermore, on 60 occasions participants appeared to wake up to answer the first EMA prompt of the day then went back to sleep resulting in non-valid activity monitor data. The results from this study indicate temporal factors contribute to EMA and ambulatory monitoring compliance and suggest the possibility of tailoring EMA prompts to either (1) avoid times when participants may be unable to answer EMA prompts (e.g., tailoring EMA prompting to sleep and wake times) or (2) strategically oversample times when participants are less likely to respond to provide more representative data (e.g., Sarker et al., 2014).

Regarding the second study objective, concurrent PA and SB were unrelated to the likelihood of answering an EMA prompt. This suggests that, for older adults, it is feasible to either stop their current activity to complete the EMA questionnaire or complete the EMA questionnaire simultaneously with their current activity. Additionally, this indicates that older adults are willing to take the loaned study phone with them while exercising. Dunton et al. (2012) previously found that normal weight adults were less likely to answer an EMA prompt while engaging in moderate-or vigorous-intensity PA and suggested that normal weight adults are more likely to engage in PA (e.g., road cycling, team sports) where answering the EMA prompt is not feasible. However, the most common form of PA for older adults is walking (Dai et al., 2015), and it is likely that walking is a behavior that is more compatible to EMA survey response.

Related to the third study objective, results indicated that when participants self-reported sitting during their current activity, SB in the 15 min before the EMA prompt did not significantly differ from the SB in the 15 min after. Individuals maintained prior levels of SB after receiving the EMA prompt, thus the EMA prompt did not encourage older adults to stand or make them feel like they should break up their SB. However, on occasions when older adults self-reported PA as their current activity, PA in the 15 min before the EMA prompt was significantly greater than PA in the 15 min after. It is possible that when older adults engaged in PA at the time of the EMA prompt, the act of reporting on their PA may have disrupted their PA behavior. Taking a break from PA to answer the EMA prompt could cause participants to reflect on their affect, physical feeling states, and cognitions (e.g., feeling tired) associated with engagement in PA and subsequently disengage with the behavior. It should be noted that although there were statistically significant differences in PA before and after the EMA prompt, that difference in time spent engaged in PA according to the ActivPAL activity monitor was less than 1 min. So, it may be that this is not a clinically or practically important difference.

Previous research addressing issues of disruptions in behavior because of, or reactivity to, EMA have produced conflicting findings depending on the population studied. For instance, in an EMA study of children’s PA, Dunton et al. (2011) found that among overweight and obese children, receiving an EMA prompt resulted in less PA after the EMA prompt compared to before the EMA prompt suggesting that EMA disrupted PA. Conversely in a EMA study of adults’ PA and SB, Dunton et al. (2012) found that there was no difference in PA before or after the EMA prompt, suggesting that individuals resumed their prior level of activity after the prompt. But there were differences in SB before and after the EMA prompt with adults engaging in more SB following the EMA prompt (Dunton et al., 2012). Differences between the results of this study and previous work may be explained by age-related differences in volume and patterns of PA and SB.

Descriptive correlations among EMA responses and concurrent PA and SB in this study were relatively similar to correlations among self-report, recalled-based questionnaires of PA and SB with objective measures via accelerometer in population based studies (e.g., Healy et al., 2011; Lee et al., 2011). However, this study was not designed to measure total volume of PA or SB as recall-based and objective measures of PA and SB commonly are, rather this study was concerned with the validity of EMA to measure current or instantaneous engagement in PA and SB.

Regarding the study objective pertaining to the validity of EMA, results indicated that device-based ActivPAL activity monitor data corresponded with EMA-reported current activity. Concurrent PA and SB in the ±15-min window around the EMA prompt were greater when participants reported engaging in PA or SB at that prompt, respectively. Consistency across EMA-reported and device-measured PA and SB suggests that older adults were truthful, aware, and accurate in reporting the nature of their current activity through the EMA questionnaires. These findings reduce concerns that older adults may report recently performed behaviors rather than their current activity, complete the EMA questionnaires haphazardly, or misrepresent their current activity due to social desirability. These results suggest that EMA represents a valid real-time data capture technique to measure PA and SB in older adults.

Although this study documents the feasibility and validity of EMA to measure current PA and SB in community-dwelling older adults, researchers should consider the cost-to-benefit ratio of employing a real-time data capture strategy such as EMA. EMA can help researchers yield innovative insights regarding PA and SB; however, costs including the (a) EMA platform or computer programmer to design an EMA application, (b) mobile phones, and (c) personnel necessary to conduct the EMA study (especially in populations with limited smartphone experience) should be weighed against the knowledge gained from this kind of study *a priori*.

The limitations of this study should be addressed. This sample was homogeneous with respect to race and ethnicity. Future research should explore the feasibility and validity of an EMA protocol among racial and ethnic minorities as there may be privacy concerns or limited proficiency with technology among specific populations of older adults (Sugie, 2016). Additionally, recruiting participants from a repository of older adults that previously participated in research may have resulted in a sample more willing to adhere to the EMA protocol. Data from this study included 5,513 EMA observations from 104 participants. While an *a priori* power analysis was not conducted, the sample size of this study (at both the occasion and person-level) is similar to that of previous EMA research studies that have been adequately powered (e.g., Dunton et al., 2011, 2012; Maher and Conroy, 2016; Maher et al., 2016; Fritz et al., 2017). Future EMA research should consider the sample size needed for adequate power *a priori*. Regarding the assessment schedule, this study employed a random signal-contingent sampling procedure which may have resulted in some PA or SB not being captured through EMA. Furthermore, participants did not report on the duration or intensity of PA or SB. However, this EMA protocol was not designed to provide a measure of total PA or SB. Rather the overall purpose of the study was to determine the feasibility and validity of sampling specific behaviors at a given moment that can then be linked with other time-intensive EMA measures such as affective states, behavioral cognitions, or physical or social context.

Ultimately, the results from this study suggest that the assessment of older adults’ PA and SB through EMA is feasible and valid. There was no evidence to suggest that older adults had difficulty answering the EMA prompts while engaged in PA or SB. Additionally, self-reported behavior through EMA corresponded with device-based measures of PA and SB. Therefore, EMA represents a significant methodological tool that can aid in our understanding of the environmental, social, and psychological processes regulating older adults’ PA and SB in the context of everyday life.

## Ethics Statement

This study was carried out in accordance with the recommendations of the University of Southern California’s Institutional Review Board with written informed consent from all subjects. All subjects gave written informed consent in accordance with the Declaration of Helsinki. The protocol was approved by the University of Southern California’s Institutional Review Board.

## Author Contributions

JM and GD contributed conception and design of the study. JM collected the data and wrote the first draft of the manuscript. JM and AR cleaned the data and performed the statistical analysis. JM, AR, and GD were involved in the interpretation of analysis. All the authors contributed to manuscript revision, read and approved the submitted version.

## Conflict of Interest Statement

The authors declare that the research was conducted in the absence of any commercial or financial relationships that could be construed as a potential conflict of interest.
